# Explorative Study on Volatile Organic Compounds of Cinnamon Based on GC-IMS

**DOI:** 10.3390/metabo14050274

**Published:** 2024-05-09

**Authors:** Yu Pan, Liya Qiao, Shanshuo Liu, Ye He, Danna Huang, Wuwei Wu, Yingying Liu, Lu Chen, Dan Huang

**Affiliations:** 1 National Engineering Research Center of Southwest Endangered Medicinal Resource Development, Guangxi Zhuang Autonomous Region Chinese Medicinal Materials Product Quality Supervision and Inspection Station, Guangxi Botanical Garden of Medicinal Plants, Nanning 530023, China; panyu@gxyyzwy.com (Y.P.); qiaoly@gxyyzwy.com (L.Q.); huangdn@gxyyzwy.com (D.H.); wuww@gxyyzwy.com (W.W.); 2 State Key Laboratory of Chinese Medicine Powder and Medicine Innovation in Hunan (Incubation), Science and Technology Innovation Center, Hunan University of Chinese Medicine, Changsha 410208, China; lss12138@stu.hnucm.edu.cn (S.L.); hyee@stu.hnucm.edu.cn (Y.H.); 3 Key Laboratory of Guangxi for High-Quality Formation and Utilization of Dao-Di Herbs, Guangxi Botanical Garden of Medicinal Plants, Nanning 530023, China; liuyy@gxyyzwy.com

**Keywords:** cinnamon, volatile organic compounds, GC-IMS, PCA, CA, PLS-DA

## Abstract

Cinnamon is one of the most popular spices worldwide, and volatile organic compounds (VOCs) are its main metabolic products. The misuse or mixing of cinnamon on the market is quite serious. This study used gas chromatography-ion migration spectroscopy (GC-IMS) technology to analyze the VOCs of cinnamon samples. The measurement results showed that 66 VOCs were detected in cinnamon, with terpenes being the main component accounting for 45.45%, followed by aldehydes accounting for 21.21%. The content of esters and aldehydes was higher in RG-01, RG-02, and RG-04; the content of alcohols was higher in RG-01; and the content of ketones was higher in RG-02. Principal component analysis, cluster analysis, and partial least squares regression analysis can be performed on the obtained data to clearly distinguish cinnamon. According to the VIP results of PLS-DA, 1-Hexanol, 2-heptanone, ethanol, and other substances are the main volatile substances that distinguish cinnamon. This study combined GC-IMS technology with chemometrics to accurately identify cinnamon samples, providing scientific guidance for the efficient utilization of cinnamon. At the same time, this study is of great significance for improving the relevant quality standards of spices and guiding the safe use of spices.

## 1. Introduction

Spices refer to a class of natural plant products with typical aromatic and spicy flavors, specifically seasonings made from dried plant seeds, buds, fruits, stems, leaves, roots, etc.—for example, pepper, cloves, and cinnamon. They can not only enhance the aroma of food but also have strong antioxidant activity and antibacterial effects [1,2] and can be used to reduce the risk of chronic diseases [3]. Spices, as a necessity of daily life, are widely used in cooking and the food industry.

Cinnamon is widely used as a spice for its fragrance and flavor in various traditional foods. It is widely used around the world. It is the dried stem bark of *Cinnamomum cassia* Presl (Fam. Lauraceae.) or *Cinnamomum verum* J. Presl. As a traditional spice and traditional Chinese medicine, it has a wide range of applications in cooking and traditional Chinese medicine. In China, cinnamon is mainly used in treating impotence, frigidity, feelings of cold and pain in the loins and knees, dyspnea in deficiency of the kidney, dizziness, inflammation of the eyes, and sore throat due to yang deficiency, precordial and abdominal pain with cold sensation, vomiting and diarrhea in deficiency cold syndrome, neurosis with a feeling of gas rushing up through the chest to the throat from the lower abdomen, amenorrhea, and dysmenorrhea [4]. Modern pharmacological research shows that cinnamon has anti-cancer effects [5,6,7]; reduces blood glucose levels in patients with type 2 diabetes and insulin resistance [8,9,10]; is antibacterial [11,12,13]; and has antioxidant [14,15], anti-inflammatory [16], anti-insomnia [17], and other pharmacological effects. Cinnamon contains volatile oils, flavonoids, sesquiterpenes, polysaccharides, and other components; volatile organic compounds (VOCs) are its main functional components [18]. Ding et al. [19] used HPLC combined with PCA and PLS-DA to evaluate the quality of the collected cinnamon bark and leaf samples. The results showed that cinnamaldehyde was the most abundant marker component, followed closely by eugenol. Pages Rebull et al. [20] conducted a study using HPLC-UV combined chemometrics (PCA, SIMCA, and PLS-DA) to identify biomarker compounds of cinnamon, such as eugenol and salicylaldehyde.

Cinnamon is distributed in China, Sri Lanka, Vietnam, and other countries. In China, the production areas are mainly concentrated in Guangxi and Guangdong provinces. The most famous cinnamons include Fangcheng Cinnamon (the Geographical Indication Certification Trademark of China), Luoding Cinnamon (the Geographical Indication Products of China), Saigon Cinnamon, and Ceylon Cinnamon. Due to different distribution regions, the corresponding ecological environment such as soil, water, temperature, and light are also different, resulting in differences in the external morphology, internal structure, and physiological and biochemical components of the product, which can lead to differences in efficacy. But genuine products and counterfeit products have similar characteristics in appearance, shape, color, and odor, which can easily cause confusion and misuse. Fake products are often mistaken for or intentionally replace genuine ones to reduce costs, and the constant occurrence of adulteration seriously affects the quality of cinnamon products. At present, the identification of cinnamon mainly adopts traditional morphological identification, physical and chemical identification, spectroscopy, chromatography, DNA barcodes, and other technologies. However, the first of these methods have strong subjectivity and are greatly affected by the environment, while the latter have problems such as complex operation, high costs, and sample pretreatment [21]. Therefore, developing accurate and rapid detection methods is of great significance for the efficient and safe use of cinnamon.

In recent years, gas chromatography-ion migration spectrometry (GC-IMS) has shown unique advantages as a new analytical method for the detection and analysis of volatile organic compounds [22,23]. Through the high separation ability of gas chromatography and the high sensitivity of ion migration spectroscopy, it can achieve a rapid and accurate analysis of complex samples. Combining chemometrics can extract useful information from the obtained complex chemical data, which helps to more efficiently identify samples [24,25,26]. As volatile organic compounds are the main source of cinnamon aroma, analyzing them using GC-IMS combined with chemometrics can help to gain a deeper understanding of the aroma components of cinnamon and provide a scientific basis for its development and utilization. There are almost no studies on the identification of VOCs in cinnamon using GC-IMS combined with chemometrics, and this difference is often one of the key factors causing differences in the quality of foods.

## 2. Materials and Methods

### 2.1. Materials

In this study, we collected four batches of cinnamon, which are Fangcheng Cinnamon (named RG-01), Luoding Cinnamon (named RG-02), Ceylon Cinnamon (named RG-03), and Saigon Cinnamon (named RG-04). Each sample is crushed into powder at a low temperature (−10 °C) and set aside for later use.

### 2.2. Analysis by GC-IMS

#### 2.2.1. Instruments and Equipment

FlavorSpec^®^ Gas Chromatograph-Ion Mobility Spectrometer from GAS (Dortmund, Germany) was used in this study.

#### 2.2.2. Sample Preparation

Sample Preparation: The cinnamon samples were crushed to approximately 100 mesh to facilitate the weighing and release of VOCs.

Weighing: 0.5 g of the sample was weighed.

Sample in headspace vial: The weighed 0.5 g of the cinnamon sample was transferred into a headspace vial, trying to distribute the sample as evenly as possible at the bottom of the vial.

Warming treatment: The headspace bottle was placed in a thermostatic water bath and the samples were allowed to warm at 80 °C for 15 min to promote the release of volatile components from the cinnamon.

Measurements: 3 parallel measurements were performed for each sample.

#### 2.2.3. Headspace Conditions

A 15 min incubation at 80 °C was followed by a non-shunt injection of 500 microliters, followed by rotating the vials at 500 revolutions per minute (rpm). It was 85 °C at the time of injection.

#### 2.2.4. GC Conditions

MXT-WAX (15 m × 0.53 mm × 1.0 m, Restek Inc., Edmond, OK, USA) was used for GC. The oven temperature was 60 °C. The carrier gas was initially 2.00 mL/min of high-purity N2 (99.999%). The flow rate increased linearly from 10.00 mL/min to 10.00 mL/min within 8 min and then was maintained for 10 min. An injection temperature of 85 °C and a run time of 59 min were used for the chromatography.

#### 2.2.5. IMS Conditions

Tritium (3H) acts as a source of ionization; a 53 mm drift tube was used; 500 V/cm electric field intensity; 45 °C drift tube temperature; N2 (99.999%) flow rate; 150 mL/min flow rate, and positive ionization mode.

### 2.3. Statistical Analysis

The calibration curves of the retention time and retention index were established, and then the retention index of the substance was calculated from the retention time of the target, and the qualitative analysis of the target was carried out using the built-in GC retention index (NIST 2020) database and the IMS migration time database of the VOCal software (from G.A.S., Dortmund, Germany, version 2.0.0). Plug-ins such as Reporter, Gallery Plot, and Dynamic PCA in VOCal data processing software (from G.A.S., Dortmund, Germany, version 2.0.0) were used to generate 3D spectra, 2D spectra, differential spectra, fingerprint spectra, and PCA plots of volatile components, respectively for comparison of VOCs between samples.

The PCA method is used for feature extraction and dimension reduction; it converts numerous variables into a small number of relevant variables, allowing a clearer understanding of categories, clusters, and outliers [27]. In PCA, the model is visualized, allowing it to be understandable and avoiding subjective judgment [28].

CA is a method of non-parametric data interpretation used in fingerprint analysis that groups samples. It displays complex data in a simple manner and is easy to use [29].

In order to observe differences between groups, a PCA-based discriminative investigation was conducted using PLS-DA in the supervised mode. PLS-DA is a statistical technique that combines PLS regression and discriminant analysis to identify differences between different sample groups and classify them in high-dimensional data. PLS-DA maximizes group differences according to previously defined classifications [30].

In addition, the predicted variable importance projection (VIP) > 1 is used as a standard to measure the influence intensity and explanatory ability of the expression pattern of the compound on the classification discrimination of each group of samples [31,32], thereby assisting in screening the main differences between samples.

## 3. Results

### 3.1. GC-IMS Analysis of VOCs in Four Cinnamon Samples

#### 3.1.1. Comparison of VOCs in Four Batches of Cinnamon

**Figure 1:**
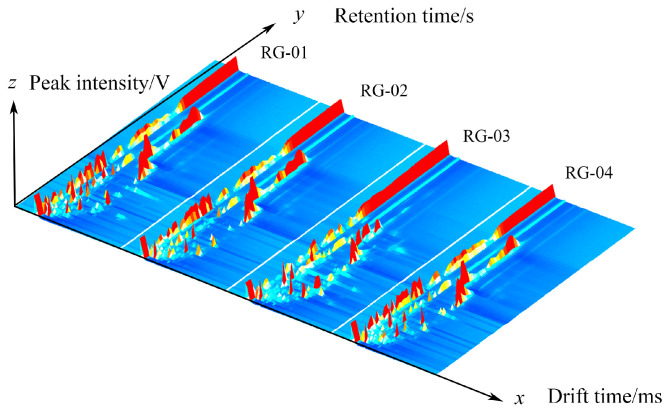
The 3D spectrum of the VOCs of four groups of cinnamon.

Figure 1 shows the 3D spectra of VOCs in cinnamon generated using the Reporter plugin program in the GC-IMS instrument analysis software (from G.A.S., Dortmund, Germany, version 2.0.0). The x, y, and z axes in the graph represent the drift time, gas chromatography retention time, and peak intensity, respectively. Each peak represents a volatile component, and the height of the red protrusion represents the strength of the component signal. The higher the red protrusion, the stronger the signal, indicating a high component content. The lower the red protrusion, the weaker the signal, indicating a low component content. In Figure 1, it can be seen that there are certain differences in four batches of cinnamon. 

**Figure 2:**
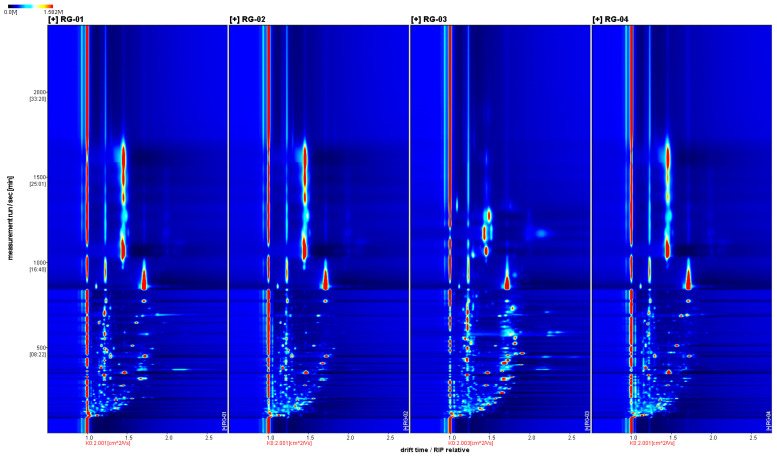
The 2D spectrum of the VOCs of four groups of cinnamon.

We further analyzed the sample’s gas phase ion migration spectrum in detail by combining the two-dimensional top view and difference plot. Figure 2 shows a two-dimensional view of VOCs in four batches of cinnamon, with the horizontal axis representing the ion drift time and the vertical axis representing the retention time. The red vertical line represents the reaction ion peak. Each bright spot represents a volatile component on either side of the reaction ion peak. In order to determine the analyte, it must be quantified, and the color depth represents the volatile compound content. The higher the content of the component, the darker the color and the larger the bright spot. Red indicates a higher content of the corresponding component, and white indicates a lower content. It is also possible to visually compare the VOCs in the four batches of cinnamon in Figure 2.

**Figure 3:**
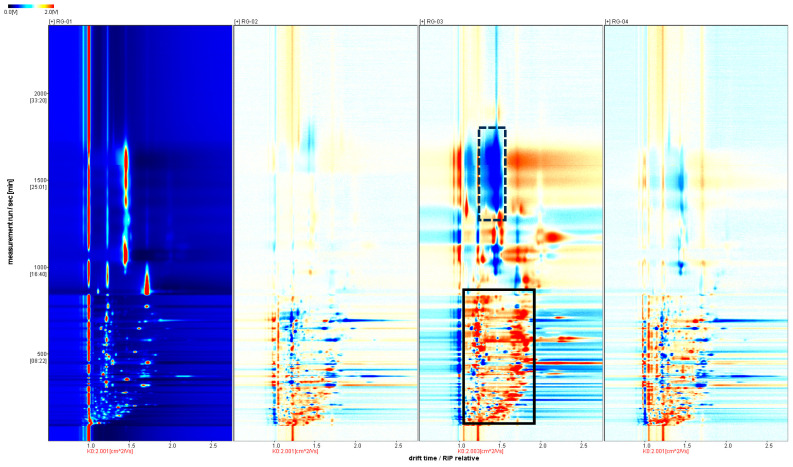
Analysis of the spectral differences between RG-01 and the other three groups of cinnamon.

As shown in Figure 3, the spectrum of the RG-01 sample is used as a reference, and the spectra of the other samples are subtracted from the reference to compare the differences between them. If the VOCs in the target sample and the reference sample are the same, the subtracted background is white, while red indicates a higher concentration of the substance in the target sample. At the same time, blue indicates a lower concentration of the substance in the target sample. We can clearly see the differences between the four batches of cinnamon through differential spectra. RG-03 has significantly less substances in the dotted black border than RG-01, and RG-01 has many red signal peaks, indicating that RG-03 has significantly more substances than RG-01.

#### 3.1.2. Qualitative Analysis of VOCs in Four Batches of Cinnamon by GC-IMS

**Figure 4:**
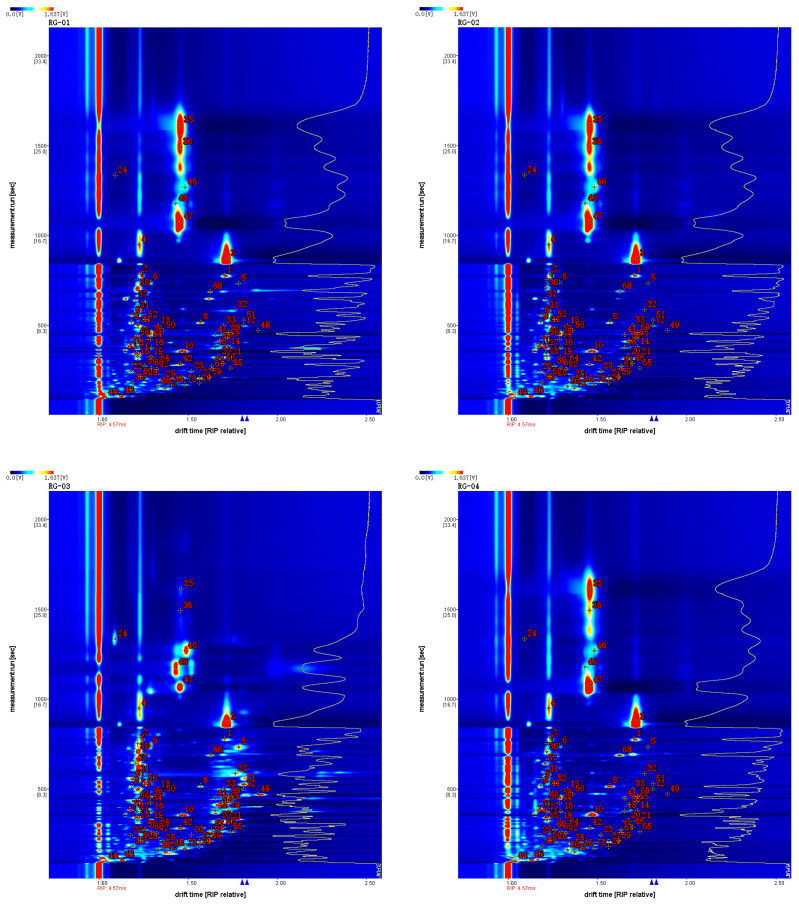
Characteristic peak position plot of VOCs of cinnamon.

In order to qualitatively compare the relative differences of the VOCs in four cinnamon samples, the Vocal plugin was used to process the data and obtain the ion migration spectra of the four cinnamon samples, as shown in Figure 4. VOCs are represented by different points in the graph, and the color depth of these points represents their concentrations. The content is higher if the color is darker, and vice versa.

Comparing NIST 2020 and IMS migration time databases for the GC retention index, it was found that a total of 66 VOCs were detected in four batches of cinnamon, mainly including 30 terpenes, 14 aldehydes, 9 esters, 6 ketones, 6 alcohols, and 1 ether. The qualitative results are shown in Table 1.

#### 3.1.3. GC-IMS Profile Analysis of VOCs in Four Batches of Cinnamon

**Figure 5:**
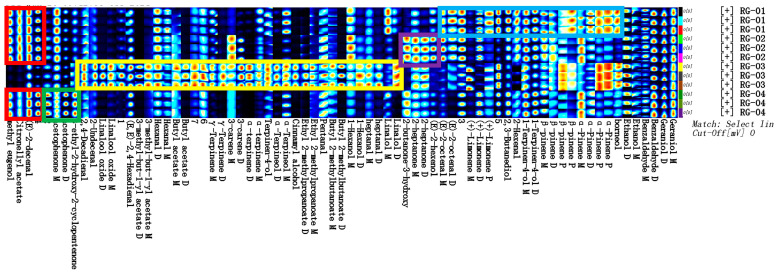
Gallery plot of VOCs selected via GC-IMS.

The construction of VOC profile maps for different varieties of cinnamon can provide an effective means for the origin traceability and quality evaluation of cinnamon. To further compare the VOCs in the samples, a profile analysis was performed on all VOCs, as shown in Figure 5. Each row represents all the signal peaks selected from a sample, and each column represents all the signal peaks of the same volatile organic compound across samples. In each grid, the higher the content, the brighter the color. Through a comparative analysis of volatile substances in samples RG-01, RG-02, RG-03, and RG-04, the results showed that, as shown in the red box, methyl eugenol, citronellate acetate, and (E)-2-decenal had higher content in RG-01, RG-02, and RG-04. As shown in the green box, acetophenone and 3-ethyl-2-hydroxy-2-cyclopentenone have higher content in RG-04. As shown in the yellow box, 2,4-decadienal, 2-decenenal, linalool oxide, (E, E)-2,4-hexadienal, 3-methyl-1-butyl acetate, hexanal, butyl acetate γ-Terpenes, 3-carene α- Terpene, terpene-4-ol α- Terpineol, cinnamyl alcohol, ethyl 2-methylpropionate, valeraldehyde, butyl 2-methylbutyrate, hexanol, heptanal, and linalool have higher content in RG-03. As shown in the purple box, 3-hydroxy-2-butanone, 2-heptanone, and (E)-2-hexenol have higher content in RG-02. As shown in the blue box, (E)-2-octenal, limonene, 2,3-butanediol, 2-hexenal, 1-terpen-4-ol β- Pinene α- Pinene, and 2-propanol have higher content in RG-01.

### 3.2. Chemometric Analysis

Chemometrics combines mathematics, statistics, computer science, and chemistry. The three chemometric methods (exploratory method and classification method) commonly used in GC-IMS data analysis are the exploratory method, represented by principal component analysis (PCA), and the classification method, represented by cluster analysis (CA) and partial least-squares discriminant analysis (PLS-DA). These methods are widely used in traditional Chinese medicine, agricultural products, food classification, medicine, etc.

#### 3.2.1. Principal Component Analysis (PCA)

**Figure 6:**
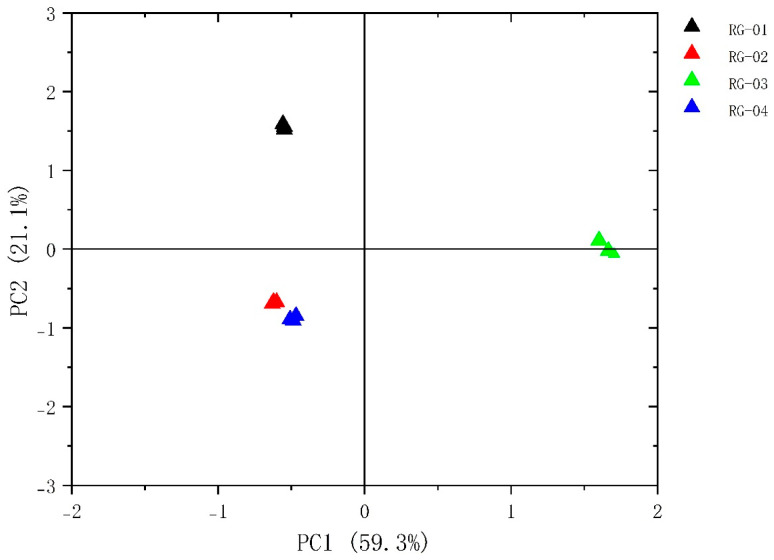
Plot of the PCA scores of VOCs in four species of cinnamon.

The PCA of VOCs in four groups of cinnamon samples, RG-01, RG-02, RG-03, and RG-04, was performed using Origin pro 2023b software. The results are shown in Figure 6. The contribution rate of PC1 is 59.3%, and the contribution rate of PC2 is 21.1%, with a cumulative contribution rate of 80.4%. The small distance between Sample 1, Sample 2, and Sample 4 indicates that their differences are small. At the same time, Sample 3 has a clear distance from the other three cinnamon groups, indicating a significant difference between Sample 3 and the other three groups of cinnamon. Certain differences exist in the content of VOCs between the four batches of cinnamon.

#### 3.2.2. Cluster Analysis (CA)

**Figure 7:**
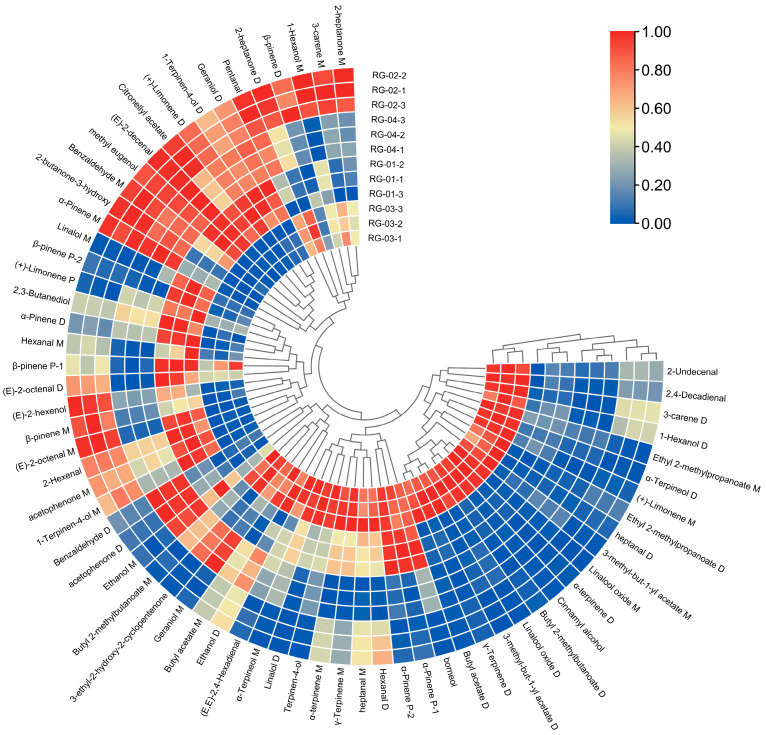
Cluster heat map of VOCs in four species of cinnamon.

Using a heatmap, data differences between groups are intuitively represented by color depth changes. A total of 66 volatile component peaks from the four groups, RG-01, RG-02, RG-03, and RG-04, were imported into TBtools software (v2.026) for CA. The results are shown in Figure 7. For the four groups of cinnamon from different origins, the clustering characteristics of cinnamon from the same origin are obvious. The clustering characteristics of the second and fourth groups are obvious, and the difference between the third group and the other three groups is significant. The elements were as follows: 2-Undecental, 2,4-Decadienal, 3-carene D, 1-Hexanol D, Ethyl 2-methylpropanoate D, Ethyl 2-methylpropanoate M γ- Terpinene D, (+)-Limene M, heptanal D, Linalool oxide M α- Terpinene D, Cinnamyl alcohol, Butyl 2-methylbutanoate D, Linalool oxide D, 3-methyl-but-1-yl acetate D, and 3-methyl-but-1-yl acetate M γ-. The content of Terpinene D and Butyl acetate D in RG-03 is higher than that of the other three groups.

#### 3.2.3. Partial Least-Squares Discriminant Analysis (PLS-DA)

**Figure 8:**
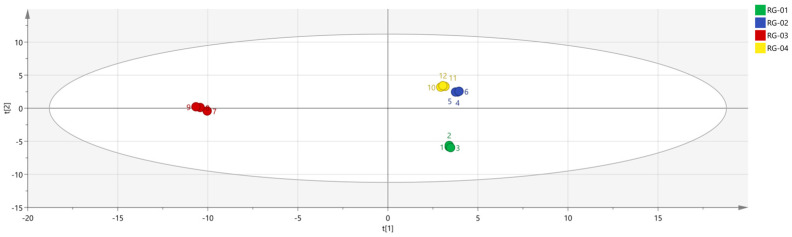
PLS−DA analysis of VOCs in four groups of cinnamon.

**Figure 9:**
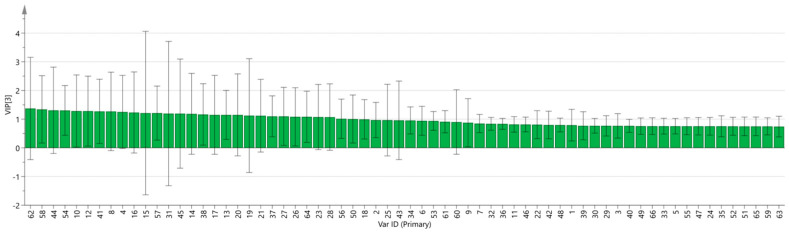
VIP diagram of the PLS-DA model.

**Figure 10:**
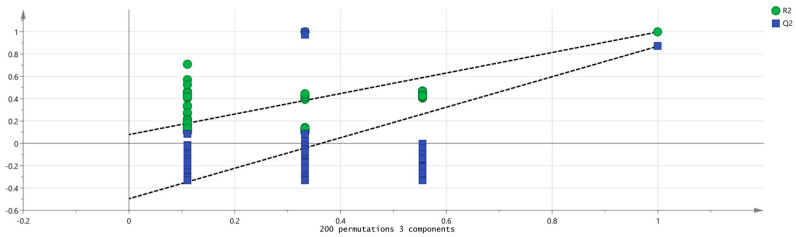
Permutation test results of VOCs in four groups of cinnamon.

SIMCA 14.1 software was used to import four groups of sample data: RG-01, RG-02, RG-03, and RG-04. The results are shown in Figure 8. The PLS-DA score chart shows that the PLS-DA model has good prediction ability (R^2^X = 0.975, R^2^Y = 0.999, Q^2^ = 0.997). The values of R^2^ and Q^2^ are close to 1, indicating that the fitting accuracy is good. RG-03 is distributed on the left side of the figure, while the other three groups of samples are all on the right side of the figure, indicating that the chemical composition of RG-03 is significantly different from other groups, which is consistent with the conclusion drawn from the PCA plot. Predicting variable importance projection VIP > 1 measures the impact of compounds on sample classification, which helps identify the main differences between samples. The higher the VIP value, the greater the effect on cinnamon. As shown in the Figure 9, 1-Hexanol M, 2-heptanone M, Ethanol M, 3-carene M, Benzaldehyde D, (E)-2-octenal D, (+)-Limonene P, acetophenone D, Geraniol M, β-pinene D, β-pinene P, 2-heptanone D, Linalol M, Pentanal, borneol, β-pinene M, (E)-2-octenal M, α-Pinene P, α-Pinene D, 3-ethyl-2-hydroxy-2-cyclopentenone, Hexanal D, 2-butanone-3-hydroxy, heptanal M, (E)-2-hexenol, Hexanal M, α-terpinene M, and Butyl 2-methylbutanoate M are the main differential index components. These compounds play an important role in distinguishing different species of cinnamon samples and are the main marker compounds. At the same time, in order to determine whether the model is overfit, we conducted 200 cross-validations to examine the R^2^ and Q^2^ values. In Figure 10, we can see that the slope of the straight line is large, indicating that the PLS-DA model does not overfit (R^2^ = 0.0784, Q^2^ = −0.496).

## 4. Discussion

In this study, we used GC-IMS to successfully analyze VOCs in cinnamon from different origins. A total of 66 VOCs were detected, including 30 terpenes, 14 aldehydes, 9 esters, and 6 ketones, as well as 6 kinds of alcohol and 1 type of ether. It can be seen that terpenes are the main components of VOCs in cinnamon, followed by aldehydes and esters. Among them, cinnamic aldehyde is an important contributor to the unique aroma and flavor of cinnamon, and it has very important pharmacological effects, such as anti-inflammatory, antiviral, and antibacterial [33,34,35]. Through the 3D spectrum, 2D spectrum, and color difference spectrum obtained by GC-IMS technology, we can clearly and intuitively see obvious differences in the VOCs in cinnamon from different origins. In addition, according to the results of the volatile component fingerprint, β-pinene and α-pinene are relatively high in the RG-01 and RG-03 samples. There are obviously more high-content substances in the RG-03 sample than in other samples. Still, the three substances, eugenol methyl ester, citronellyl acetate, and (E)-2-decenal, have the lowest content in the RG-03 sample, while they are higher in the other samples. 

To analyze the chemical information obtained, we used chemometrics. The PCA method is commonly used in model recognition to identify similar samples and is combined with CA and PLS-DA for feature-based classification of samples [36]. By analyzing the PCA results, PCA1 and PCA2 were 59.3% and 21.1%, respectively, with a cumulative contribution rate of 80.4%, indicating that the two principal components can better reflect the original data. The reflected information, the PCA results, confirmed that the differences between the RG-01, RG-02, and RG-04 samples are relatively small, while the RG-03 sample is far from the other three samples, and there are obvious differences between them. In addition, according to the results of heat map clustering and PLS-DA analysis, we can also clearly observe that RG-01, RG-02, and RG-04 samples are closer to the same category; this could be explained by the fact that the three cinnamon cultivation areas are not far apart. The RG-03 sample is obviously different from them, further verifying the previous conclusion. This may be closely related to the geographical environment in which they grow. RG-03 grows in sunny tropical regions, while other cinnamons mostly grow in subtropical regions. The VIP results show that volatile substances such as 1-Hexanol M, 2-heptanone M, and Ethanol M in the cinnamon samples have the greatest impact on the flavor of the cinnamon samples.

Cinnamon is an important spice in cooking and can provide a unique aroma. However, there are also some instances of inferior or adulterated cinnamon on the market, which may harm the quality and safety of the product. Therefore, accurately identifying cinnamon from different origins is one of the most important steps to ensuring product quality and safety. Currently, the quality evaluation methods for cinnamon mainly include HPLC, GC, NIR, and GC-MS technologies [37,38,39]. As a fast and non-destructive technology with higher sensitivity and resolution, GC-IMS can detect lower concentrations of VOCs and has broad application prospects in cinnamon quality control, authenticity identification, and aroma analysis. This study used this technology to measure the content of VOCs in cinnamon from different origins. It established a more convenient and effective method for identifying the spice cinnamon, enabling more efficient and safe utilization of cinnamon resources in food, spices, and medicine.

## 5. Conclusions

This study used GC-IMS technology combined with chemometrics to analyze and compare VOCs in various cinnamon samples. A total of 66 VOCs were detected, including 30 terpenes, 14 aldehydes, 9 esters, 6 alcohols, 6 ketones, and 1 ether. Terpenes account for 45.45%, while aldehydes account for 21.21%. Therefore, terpenes and aldehydes are the most abundant VOCs in cinnamon. The results of fingerprint analysis combined with PCA, CA, PLS-DA, and other data indicate that the ester and aldehyde contents in RG-01, RG-02, and RG-04 are relatively high; RG-01 has a higher alcohol content, RG-02 has a higher ketone content, and RG-03 has a higher ester content. The visualized data indicates that there will be certain differences in the VOCs in various cinnamon samples.

This study developed a simple and efficient GC-IMS method for the identification of VOCs in cinnamon. Compared with traditional identification methods, this method overcomes the problems of poor specificity and relatively low accuracy. A comprehensive and systematic analysis was conducted on the VOCs in cinnamon, revealing the types of aroma components and providing strong support for the efficient utilization of cinnamon. At the same time, it also provides a reference for the identification of other spices.

## Figures and Tables

**Table 1:** Results of component analysis of VOCs in cinnamon.

No	Compounds	CAS	Molecular Formula	MW	RI	Rt/s	Dt/ms
1	1-Terpinen-4-ol D	C562743	C_10_H_18_O	154.3	1180.1	777.504	1.70722
2	1-Terpinen-4-ol M	C562743	C_10_H_18_O	154.3	1182	783.315	1.23744
3	Geraniol D	C106241	C_10_H_18_O	154.3	1210.3	871.554	1.72796
4	Geraniol M	C106241	C_10_H_18_O	154.3	1232.7	948.221	1.22722
5	α-Terpineol D	C98555	C_10_H_18_O	154.3	1164.3	732.687	1.78782
6	α-Terpineol M	C98555	C_10_H_18_O	154.3	1167.5	741.366	1.29145
7	Terpinen-4-ol	C562743	C_10_H_18_O	154.3	1171	751.492	1.21992
8	Acetophenone D	C98862	C_8_H_8_O	120.2	1072	517.153	1.57321
9	Acetophenone M	C98862	C_8_H_8_O	120.2	1072	517.153	1.18488
10	Benzaldehyde D	C100527	C_7_H_6_O	106.1	970.9	356.587	1.4681
11	Benzaldehyde M	C100527	C_7_H_6_O	106.1	970.9	356.587	1.14984
12	(E)-2-Octenal D	C2548870	C_8_H_14_O	126.2	1062.3	498.662	1.81392
13	(E)-2-Octenal M	C2548870	C_8_H_14_O	126.2	1061.6	497.283	1.33401
14	β-Pinene P	C127913	C_10_H_16_	136.2	988.7	378.791	1.72682
15	β-Pinene P	C127913	C_10_H_16_	136.2	988.3	378.347	1.65397
16	β-Pinene D	C127913	C_10_H_16_	136.2	985.9	375.235	1.29899
17	β-Pinene M	C127913	C_10_H_16_	136.2	983.8	372.568	1.22
18	α-Pinene M	C80568	C_10_H_16_	136.2	943.3	324.558	1.21897
19	α-Pinene D	C80568	C_10_H_16_	136.2	942.5	323.669	1.29489
20	α-Pinene P	C80568	C_10_H_16_	136.2	943.3	324.558	1.67552
21	α-Pinene P	C80568	C_10_H_16_	136.2	940.5	321.447	1.73708
22	2-Hexenal	C505577	C_6_H_10_O	98.1	851.7	242.32	1.18101
23	(E)-2-Hexenol	C928950	C_6_H_12_O	100.2	853	243.209	1.52265
24	Cinnamyl Alcohol	C104541	C_9_H_10_O	134.2	1323.3	1334.331	1.09418
25	2,3-Butanediol	C513859	C_4_H_10_O_2_	90.1	783.1	198.295	1.36304
26	2-Butanone-3-Hydroxy	C513860	C_4_H_8_O_2_	88.1	709.4	159.656	1.33619
27	Hexanal D	C66251	C_6_H_12_O	100.2	798.5	207.446	1.56833
28	Hexanal M	C66251	C_6_H_12_O	100.2	796.8	206.429	1.26513
29	Ethyl 2-Methylpropanoate D	C97621	C_6_H_12_O_2_	116.2	744.3	176.942	1.56675
30	Ethyl 2-Methylpropanoate M	C97621	C_6_H_12_O_2_	116.2	754	182.026	1.19406
31	Linalol M	C78706	C_10_H_18_O	154.3	1103.6	582.614	1.21977
32	Linalol D	C78706	C_10_H_18_O	154.3	1104.4	584.504	1.76789
33	γ-Terpinene D	C99854	C_10_H_16_	136.2	1062.4	498.83	1.70206
34	γ-Terpinene M	C99854	C_10_H_16_	136.2	1064.4	502.61	1.2238
35	Methyl Eugenol	C93152	C_11_H_14_O_2_	178.2	1373.9	1615.044	1.46374
36	Citronellyl Acetate	C150845	C_12_H_22_O_2_	198.3	1352.9	1491.955	1.46092
37	3-Ethyl-2-Hydroxy-2-CyclopeNtenone	C21835018	C_7_H_10_O_2_	126.2	1129.1	641.552	1.20911
38	Borneol	C507700	C_10_H_18_O	154.3	1155	707.475	1.21716
39	(+)-Limonene D	C138863	C_10_H_16_	136.2	1036.3	452.134	1.29163
40	(+)-Limonene M	C138863	C_10_H_16_	136.2	1030.9	443.002	1.2188
41	(+)-Limonene P	C138863	C_10_H_16_	136.2	1033.3	446.915	1.65583
42	(E,E)-2,4-Hexadienal	C142836	C_6_H_8_O	96.1	903.8	283.739	1.46049
43	Ethanol D	C64175	C_2_H_6_O	46.1	509.1	106.67	1.13303
44	Ethanol M	C64175	C_2_H_6_O	46.1	468.1	98.64	1.04239
45	Pentanal	C110623	C_5_H_10_O	86.1	730.3	169.806	1.41087
46	2-Undecenal	C2463776	C_11_H_20_O	168.3	1309.9	1268.716	1.48683
47	(E)-2-Ecenal	C3913813	C_10_H_18_O	154.3	1266.7	1077.838	1.46558
48	2,4-Decadienal	C2363884	C_10_H_16_O	152.2	1290.1	1177.253	1.43466
49	Butyl 2-Methylbutanoate D	C15706737	C_9_H_18_O_2_	158.2	1046.8	470.298	1.89602
50	Butyl 2-Methylbutanoate M	C15706737	C_9_H_18_O_2_	158.2	1046.8	470.298	1.3633
51	Linalool Oxide D	C60047178	C_10_H_18_O_2_	170.3	1077.7	528.43	1.81683
52	Linalool Oxide M	C60047178	C_10_H_18_O_2_	170.3	1078.7	530.405	1.26283
53	3-Carene D	C13466789	C_10_H_16_	136.2	1012.7	413.543	1.67851
54	3-Carene M	C13466789	C_10_H_16_	136.2	1016.5	419.463	1.29223
55	α-Terpinene D	C99865	C_10_H_16_	136.2	1022	428.342	1.72448
56	α-Terpinene M	C99865	C_10_H_16_	136.2	1022.5	429.177	1.21953
57	2-Heptanone D	C110430	C_7_H_14_O	114.2	892.4	272.936	1.63499
58	2-Heptanone M	C110430	C_7_H_14_O	114.2	893	273.436	1.25997
59	Butyl Acetate D	C123864	C_6_H_12_O_2_	116.2	804.2	210.924	1.62396
60	Butyl Acetate M	C123864	C_6_H_12_O_2_	116.2	806.2	212.174	1.23712
61	1-Hexanol D	C111273	C_6_H_14_O	102.2	869.4	255.183	1.63814
62	1-Hexanol M	C111273	C_6_H_14_O	102.2	867.1	253.432	1.32772
63	Heptanal D	C111717	C_7_H_14_O	114.2	905.8	285.688	1.70038
64	Heptanal M	C111717	C_7_H_14_O	114.2	906.3	286.189	1.33166
65	3-Methyl-but-1-yl acetate D	C123922	C_7_H_14_O_2_	130.2	876.7	260.684	1.74214
66	3-Methyl-but-1-yl acetate M	C123922	C_7_H_14_O_2_	130.2	877.4	261.184	1.29936

## Data Availability

The data presented in this study are available upon reasonable request from the corresponding author. The data are not publicly available due to privacy restrictions.
